# Mitochondrial Phylogenomics of Tenthredinidae (Hymenoptera: Tenthredinoidea) Supports the Monophyly of Eriocampinae stat. nov.

**DOI:** 10.3390/biology15020202

**Published:** 2026-01-22

**Authors:** Siying Wan, Xiao Li, Beibei Tan, Meicai Wei, Gengyun Niu

**Affiliations:** Laboratory of Insect Systematics and Evolutionary Biology, College of Life Sciences, Jiangxi Normal University, Nanchang 330022, China; wsy9594@126.com (S.W.); lixiao202309@163.com (X.L.); beibeitan2020@126.com (B.T.)

**Keywords:** Tenthredinidae, Eriocampinae stat. nov., mitochondrial genome, phylogenetic analysis, tRNA rearrangement, divergence time estimation

## Abstract

Tenthredinidae (Hymenoptera: Tenthredinoidea), the most species-rich sawfly family, features a controversial subfamily classification, particularly regarding *Eriocampa* Hartig, 1837, *Conaspidia* Konow, 1898, and their relatives. In this study, we sequenced and characterized 15 complete mitochondrial genomes from *Eriocampa*, *Eriocampopsis* Takeuchi, 1952, and *Conaspidia*, and reconstructed the phylogeny of Tenthredinidae using a mitogenomic dataset including 69 species from 16 subfamilies. The mitochondrial genomes of these genera exhibited genus-specific tRNA rearrangements within the IQM and ARNS1EF clusters. Phylogenetic analyses using both Maximum Likelihood and Bayesian Inference consistently recovered (*Eriocampa* + *Eriocampopsis* + *Conaspidia*) as a monophyletic lineage distinct from other subfamilies of Tenthredinidae. Divergence-time estimates indicate that the *Eriocampa* lineage diverged from other tenthredinids around the Late Cretaceous–Paleocene boundary (~70 Ma) and diversified during the Eocene. This timing coincides with the radiation of their host plants (Araliaceae, Betulaceae, and Juglandaceae). We also compared the morphology of Eriocampinae with that of other subfamilies of Tenthredinidae and summarized the diagnostic characters of Eriocampinae. Integrating morphological and mitogenomic evidence supports the recognition of Eriocampinae Rohwer, 1911 stat. nov. This study not only clarifies the phylogenetic position of these genera but also provides new insights into the coevolutionary history between sawflies and angiosperms.

## Simple Summary

Tenthredinidae, the most diverse family of ‘Symphyta’ (the basal lineages of Hymenoptera), has long been taxonomically unstable at the subfamily level. The systematic positions of *Eriocampa* Hartig, 1837, *Conaspidia* Konow, 1898, and their relatives have remained controversial for over a century. In this study, we sequenced and analyzed the complete mitochondrial genomes of 15 species representing *Eriocampa*, *Eriocampopsis* Takeuchi, 1952, and *Conaspidia*. Phylogenetic reconstruction using mitogenomic data from 69 sawfly species revealed that these three genera form a distinct, well-supported monophyletic clade separate from other subfamilies of Tenthredinidae. Integrating evidence from mitogenomic rearrangements, morphological characters, and divergence-time estimates strongly supports the recognition of Eriocampinae Rohwer, 1911 stat. nov. as a distinct subfamily. Divergence analyses indicate that the radiation of Eriocampinae is closely correlated with the diversification of their host plants in the families Araliaceae, Betulaceae, and Juglandaceae, spanning the Late Cretaceous to Paleogene periods. This study provides robust molecular and temporal evidence for the recognition of this new subfamily, offering a refined framework for future phylogenetic and evolutionary studies of Tenthredinidae.

## 1. Introduction

Tenthredinidae, the largest and most widely distributed family in the superfamily Tenthredinoidea (Hymenoptera), occurs in all zoogeographical regions except Antarctica. Southeast Asia, especially China, represents one of its major centers of diversity. The larvae are primarily phytophagous, feeding on leaves, although some lineages exhibit specialized habits such as leaf mining, gall induction, or stem boring. Many species are economically important pests in forestry, orchards, and ornamental plants [1]. The limited dispersal ability of adults, coupled with strong host specificity and frequent host shifts, has promoted extensive speciation and diversification, particularly among lineages associated with angiosperms [2].

Despite its taxonomic richness, the phylogenetic relationships within Tenthredinidae remain controversial, especially concerning the placement of certain tribes and genera. Among these, the systematic status of the tribe Eriocampini Rohwer, 1911, which includes *Eriocampa* Hartig, 1837, *Eriocampopsis* Takeuchi, 1952, and a peculiar genus, *Conaspidia* Konow, 1898, has long been debated. Previous studies have variably placed these taxa within Tenthredininae or Allantinae, reflecting persistent uncertainty and disagreement among researchers [3,4,5].

In recent decades, both morphological and molecular approaches have been applied to clarify the phylogenetic relationships within Tenthredinidae, yet neither has consistently resolved the phylogeny of Eriocampini. Morphological analyses based on characters such as the ovipositor, head, thorax, and wings failed to support the monophyly of Tenthredinidae or to establish stable relationships among its subfamilies [6,7]. Likewise, molecular phylogenies based on nuclear protein-coding genes, fossil-calibrated chronograms, diversification models, and large-scale phylogenomic datasets (e.g., UCEs) have not yielded well-supported placements for Eriocampini [2,8,9].

Recent mitochondrial evidence has provided additional insights. Based on 13 mitochondrial protein-coding genes (13 PCGs) from 40 species, Liu et al. (2022) recovered *Conaspidia* and *Eriocampa* as sister groups, together forming a basal lineage of Tenthredinidae with Selandriinae [10]. Traditional comparative morphological studies, however, have shown considerable disagreement in determining the systematic positions of specific genera due to insufficient supporting evidence. With the rapid development of molecular systematics, analyses based on genomic data have provided more reliable evidence for resolving these taxonomic uncertainties [11,12]. Nevertheless, despite these methodological advances, the systematic position of Eriocampini remains unresolved, highlighting the need for further investigation using mitogenomic data and broader taxon sampling. The genus *Eriocampa*, which is distributed mainly in the Holarctic region and especially abundant in eastern Asia [13], exemplifies such taxonomic ambiguity. Its taxonomic position has been the subject of considerable disagreement across studies. Ashmead (1898) assigned *Eriocampa* to Selandriinae (then treated as part of Selandriidae) [14], while later studies placed it in Heterarthrinae or Blennocampinae within Tenthredinidae [4,5,15], or even in Caliroinae of Heterarthridae [16]. Subsequent authors proposed additional alternatives, situating *Eriocampa* within Allantinae [17], Tenthredininae [16], or within complex tribal systems such as Eriocampini under Selandriinae [18]. These discrepancies illustrate the persistent instability of its classification.

Similarly, the genus *Conaspidia*, which is distributed in eastern Asia [5,19,20], exhibits uncertain systematic status. It has been variously assigned to Sioblini s. lat. [21], Sioblini s. str. [16], or even to a distinct tribe, Conaspidini, under Sioblinae [22]; more recent studies placed it in Tenthredininae [5].

To clarify these long-standing taxonomic ambiguities, the present study reconstructs a mitochondrial phylogeny of Tenthredinidae, including 69 species in total—55 representing 16 subfamilies of the ingroup and 14 species from 8 families as outgroups (Table S1). Special attention is given to 15 species of *Eriocampa* (5 species), *Eriocampopsis* (one species), and *Conaspidia* (9 species), providing new insights into their evolutionary relationships and systematic positions within the family.

## 2. Materials and Methods

### 2.1. Samples Analyzed

Specimens of 15 species were provided by Jiangxi Normal University, China, and the specimen data are presented in Table S2. Total genomic DNA was isolated from the hind leg of each ethanol-preserved specimen using the DNeasy Tissue Kit (Qiagen, Hilden, Germany), following the manufacturer’s instructions. The extracted genomic DNA was initially assessed for concentration and purity using a spectrophotometer (SpectroDrop™, Maestrogen, Inc., Waltham, MA, USA), and subsequently quantified accurately using a Qubit fluorometer (Thermo Fisher Scientific, Waltham, MA, USA) before library construction.

### 2.2. Mitogenome Sequencing, Annotation, and Analyses

Library preparation and sequencing. Sequencing libraries (~350 bp insert size) were prepared with Illumina TruSeq-compatible adapters (Novogene, Beijing, China) and quantified with an Agilent 2100 Bioanalyzer (Agilent Technologies, Santa Clara, CA, USA). High-throughput sequencing was performed on two Illumina platforms: HiSeq 4000 (Shanghai Majorbio Bio-pharm Technology Co., Ltd., Shanghai, China) and NovaSeq 6000 (Novogene, Beijing, China), both generating 150 bp paired-end reads. Raw reads are quality-checked with FastQC v0.12.0 and trimmed using BBDuk in Geneious R11 (Biomatters Ltd., Auckland, New Zealand) [23,24]; low-quality reads (<100 bp) and duplicates were removed.

Genome annotation. Clean reads were assembled de novo with MIRA in Geneious R11 (Biomatters Ltd., Auckland, New Zealand), and contigs were further refined by reference-guided mapping at medium–low sensitivity. Final assemblies were manually curated to obtain complete mitochondrial genomes. The boundaries and locations of protein-coding genes (PCGs) and rRNA genes were identified by comparing the reported tenthredinid homologous gene sequences using ORF Finder (https://www.ncbi.nlm.nih.gov/orffinder/, accessed on 10 July 2025) and by homology-based BLAST (https://blast.ncbi.nlm.nih.gov/, accessed on 9 January 2026) searches in GenBank (https://www.ncbi.nlm.nih.gov/genbank/, accessed on 9 January 2026). The precise ends of rRNA genes were predicted from the boundaries of the neighboring tRNA genes. The 15 mitogenome sequences have been uploaded to GenBank, and their accession numbers are provided in Table S1.

Sequence analysis. Summary statistics on base composition, nucleotide substitution, and codon usage were analyzed with MEGA v11.0.13 [25]. Strand asymmetries were calculated using the formulae: AT skew = (A − T)/(A + T) and GC skew = (G − C)/(G + C) [26].

### 2.3. Phylogenetic Analysis

Alignment and model selection. Fifty-five species from 16 subfamilies of Tenthredinidae were included as the ingroup, with 14 species from eight other families as outgroups (Table S1). Thirty-three previously reported tenthredinoid mitogenomes were obtained from GenBank. Each PCG was aligned using codon-based multiple alignment with MAFFT v5.3, as implemented in the TranslatorX server (http://161.111.160.230/index_v5.html, accessed on 9 January 2026). The RNA genes were aligned as DNA using the MAFFT algorithm as implemented in Geneious R11 [24,27]. Each resulting alignment was concatenated using Phylosuite v.1.2.3 [28]. Maximum likelihood (ML) analyses were performed with IQ-TREE (http://iqtree.cibiv.univie.ac.at/, accessed on 9 January 2026) using the GTR+G+I substitution model. Branch support was assessed with 1000 ultrafast bootstrap replicates (UFBoot) and 1000 SH-aLRT replicates. The number of computational threads was automatically determined with the “-nt AUTO” option.

Phylogenetic inference. Phylogenetic analyses were conducted using two datasets: (i) the nucleotide sequences of the 13 protein-coding genes (PCGs) (11,849 bp), and (ii) the concatenated sequences of the 13 PCGs and two rRNAs (16,144 bp). Maximum likelihood (ML) analyses were performed with IQ-TREE (http://iqtree.cibiv.univie.ac.at/, accessed on 10 July 2025) using the GTR+G+I substitution model. Branch support was assessed with 1000 ultrafast bootstrap replicates (UFBoot) and 1000 SH-aLRT replicates. The number of computational threads was automatically determined with the “-nt AUTO” option. For Bayesian inference (BI) phylogenetic reconstruction, the best-fitting nucleotide substitution model (GTR+F+I+G4) was first determined using ModelFinder [29]. Subsequent analyses were performed in MrBayes v3.2 with eight independent chains run for 500,000 generations, sampling every 100 generations, and discarding the initial 10% of samples as burn-in to ensure convergence and stability of the results [30].

Divergence time estimation. Divergence time estimation was conducted using the MCMCTree program implemented in the PAML v4.9j package [31]. The topology inferred from IQ-TREE was used as the constrained tree. An independent rates model was applied, with the GTR+G+I nucleotide substitution model. Fossil calibrations were assigned to several key nodes based on previous studies and fossil records (Table S3). In this study, three fossil calibration points were used: *Palaeathalia laiyangensis* Zhang, 1985, Hymenoptera, Athaliidae [32] (minimum 113 Ma, maximum 125 Ma), *Cenocimbex menatensis* Nel, 2005, Hymenoptera, Cimbicidae [33] (minimum 59.24 Ma, maximum 61.66 Ma), and *Eriocampa tulameenensis* Rice, 1968, Hymenoptera, Tenthredinidae [34] (minimum 47.8 Ma, maximum 56 Ma). All temporal constraints were implemented as soft bounds, and the corresponding prior probability distributions were calculated automatically by MCMCTree (PAML v4.9j) [31]. The Markov chain Monte Carlo (MCMC) analysis was run for 1,800,000 generations, with the first 300,000 discarded as burn-in. Sampling was performed every 10 generations, yielding 150,000 effective samples [35,36,37]. Two independent runs were performed to check convergence, and Tracer v1.7.2 was used to evaluate the effective sample sizes (ESS > 200) [38]. Divergence time estimates were obtained by combining the posterior distributions from the two independent runs and were visualized in the time-calibrated phylogeny.

## 3. Results

### 3.1. Mitogenome Architectures and Nucleotide Compositions

**Figure 1:**
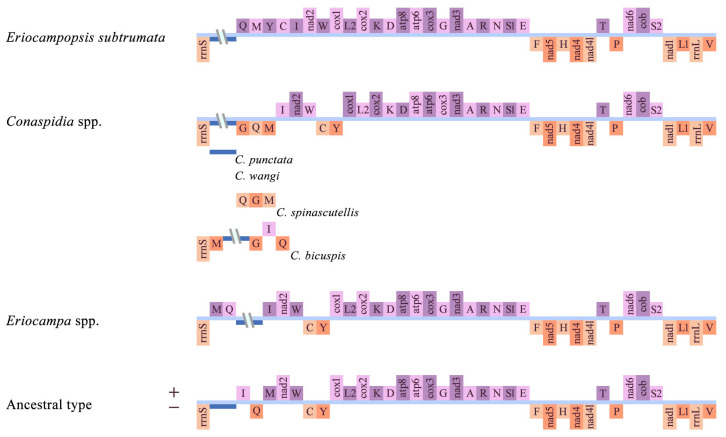
Structure of the Eriocampinae mitochondrial genome in reference to the ancestral insect mitochondrial genome type. Genes on the J-strand (+) and N-strand (−) are indicated in purple and orange, respectively. The A+T-rich region is shaded in blue, and tRNA genes are identified by their corresponding single-letter amino acid codes.

The complete mitogenomes of 15 species representing three genera were sequenced and characterized: *Eriocampa dentelle* Nie & Wei, 2011 (15,095 bp), *E. nitiditerga* Wei, 2025 (15,104 bp), *E. ovata* Linné, 1760 (15,083 bp), *E. eradia* sp. nov. (15,105 bp), *E. rufomaculata* Nie & Wei, 1998 (15,208 bp), *Eriocampopsis subtrumata* Takeuchi, 1952 (15,182 bp), *Conaspidia jiaoae* Wei, 2025 (two specimens; 15,221 bp and 15,250 bp), *C. indistincta* Wei, 1997 (15,208 bp), *C. punctata* Wei, 1997 (15,834 bp), *C. major* Wei, 1997 (15,327 bp), *C. spinascutellis* Wei, 1997 (16,163 bp), *C. wangi* Wei, 2015 (15,924 bp), *C. bicuspis* Malaise, 1945 (15,730 bp), and *C. zhoui* sp. nov. (15,317 bp). Each mitogenome (Figure 1, Table S4) comprised a typical set of 37 genes: 13 PCGs, 22 tRNAs, two rRNAs, and an AT-rich region. The observed length variation among these mitogenomes was primarily due to variation in the A+T-rich region (Table S4). Most genes were located on the majority (J) strand, except for four PCGs (*nad1*, *nad4*, *nad4l*, and *nad5*), two rRNAs, and a small number of tRNAs. Among the 15 analyzed species representing three genera, the mitochondrial gene orders showed distinct rearrangement patterns. Compared with the inferred ancestral pancrustacean mitogenome (*Daphnia pulex*), all mitogenomes exhibited several tRNA gene rearrangements in the region upstream of *nad2* (Figure 1). The previously considered relatively conserved gene block (*cox3*–*trnG*–*nad3*) underwent remote inversion across all species of *Conaspidia*. Specifically, *trnG* was rearranged from the original *cox3*–*trnG*–*nad3* gene cluster to the *trnI*–*trnQ*(-)–*trnM* cluster, but its exact position varied among species: in *C. spinascutellis*, the order was *trnQ*(-)–*trnG*(-)–*trnM*(-)–*trnI*; in *C. bicuspis*, the order was *trnM*(-)–*trnG*(-)–*trnI*–*trnQ*(-); whereas in other *Conaspidia* species, the consistent arrangement was *trnG*(-)–*trnQ*(-)–*trnM*(-)–*trnI* (Figure 1). In contrast, the rearrangements in *Eriocampa* mainly occurred within the *trnI*–*trnQ*(-)–*trnM* cluster, where *trnM* was translocated downstream of rrnS(-), and trnQ underwent a remote inversion, relocating it upstream of the control region (Figure 1). For *Eriocampopsis*, the rearrangements were primarily concentrated in the tRNA gene region upstream of nad2, where *trnQ*, *trnC*, and *trnY* experienced remote inversions. At the same time, *trnI* and *trnM* were translocated, ultimately resulting in the order *trnQ*–*trnM*–*trnY*–*trnC*–*trnI*–*nad2*–*trnW*–*cox1* (Figure 1).

We successfully sequenced and annotated the complete mitochondrial genomes of 15 representative species from three genera. All genomes exhibited the typical circular, double-stranded structure, ranging from 15,083 bp (*E. ovata*) to 16,163 bp (*C. spinascutellis*), and contained the standard set of 37 genes, including 13 protein-coding genes (PCGs), 22 tRNAs, two rRNAs, and one control region (Table S4). The nucleotide compositions were strongly A+T-biased, ranging from 79.3% (*E. eradia*) to 82.3% (*C. zhoui*) and averaging ~80.7%. AT-skew values varied between 0.0085 and 0.074, while GC-skew values ranged from −0.246 to −0.164, reflecting the strand-specific nucleotide usage typical of insect mitogenomes (Table S4). Relative synonymous codon usage (RSCU) analyses showed a pronounced preference for codons ending with A or U (Figure S1; Table S5). The most frequently used codons were UUA (Leu, RSCU = 5.11), AUU (Ile, RSCU = 1.88), and UUU (Phe, RSCU = 1.84), whereas codons ending with C or G were generally underrepresented. The majority of PCGs initiated with ATG, ATA, or ATT, although non-canonical start codons such as TTG and GTG were also identified. Stop codons were mainly TAA and TAG; however, incomplete stop codons (T– or TA–) were observed in several genes (e.g., *nad1*, *nad2*, *nad4*), which are presumed to be completed by post-transcriptional polyadenylation.

### 3.2. Phylogeny of Tenthredinidae

**Figure 2:**
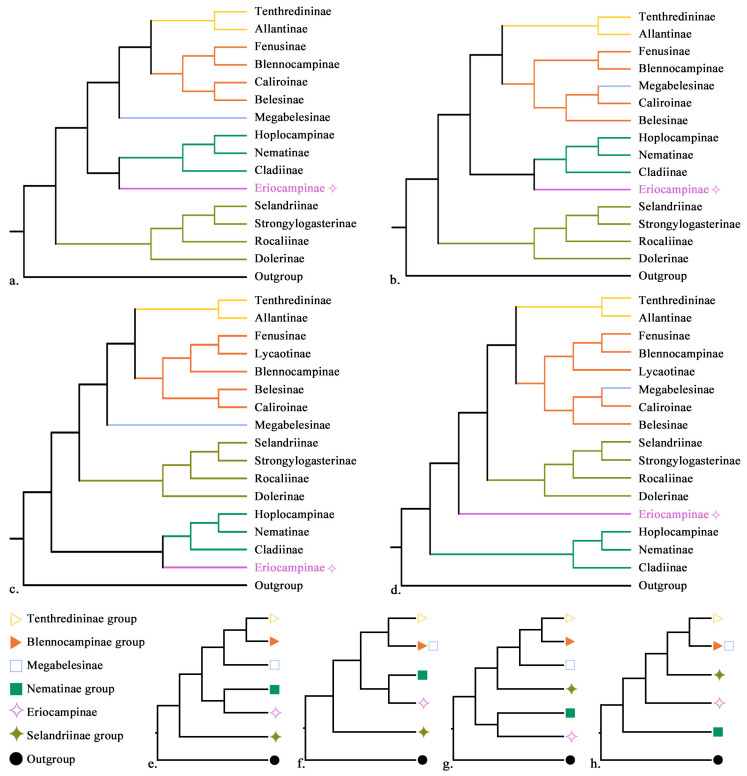
Phylogenetic trees of Tenthredinidae inferred from mitochondrial datasets under maximum likelihood (ML) and Bayesian inference (BI) analyses. Panels (**a**–**d**) display topologies based on the 13 protein-coding genes (13PCGs) and 13PCGs + rRNA datasets, with subfamily names indicated at the branch terminals: (**a**) 13PCGs_ML, (b) 13PCGs_BI, (**c**) 13PCGs + rRNA_ML, and (**d**) 13PCGs + rRNA_BI. Panels (**e**–**h**) show the corresponding topologies labeled with subfamily group names: (**e**) 13PCGs_ML, (**f**) 13PCGs_BI, (**g**) 13PCGs + rRNA_ML, and (**h**) 13PCGs + rRNA_BI. Colors representing different subfamily groups are shown in the lower left corner: yellow, Tenthrediniinae group; orange, Blennocampinae group; light blue, Megabelesinae; green, Nematinae group; purple, Eriocampinae; olive, Selandriinae group; and black, outgroup.

In this study, two datasets (13 PCGs and 13 PCGs + rRNA) were analyzed using Bayesian inference (BI) and maximum likelihood (ML), yielding four alternative phylogenetic topologies (Figure 2). Overall, both dataset composition and inference method influenced tree topology, with ML analyses generally showing lower branch support than the corresponding BI analyses.

Despite these differences, several major patterns were consistently recovered. All ML analyses identified five major lineages and one independent clade within Tenthredinidae, and both ML and BI trees strongly supported the sister-group relationship of ((*Conaspidia* + *Eriocampopsis*) + *Eriocampa*) with the Nematinae group. This clade was consistently separated from the traditional subfamilial placements in Selandriinae, Heterarthrinae, Allantinae, and Tenthredininae, with strong statistical support (PP = 1, ML ≥ 99%) [3,4,5,14,16] (Figure S2).

The relative placement of major subfamilial groups varied between datasets. In analyses based on the 13 PCGs dataset, both BI and ML trees recovered the Selandriinae group as the basal lineage of Tenthredinidae. In contrast, analyses of the 13 PCGs + rRNA dataset yielded alternative basal placements: ML analyses placed the (((*Conaspidia* + *Eriocampopsis*) + *Eriocampa*) + Nematinae group) clade at the base, whereas BI analyses supported the Nematinae group (((Hoplocampinae + Nematinae) + Cladiinae)) as the basal lineage (Figure 2).

Within the Tenthredininae group, Tenthredininae and Allantinae were consistently supported as sister taxa across all analyses (PP ≥ 0.97, ML ≥ 97%). However, their relationships with other subfamilies varied depending on dataset and inference method. In ML analyses of the 13 PCGs dataset, this lineage was recovered as sister to ((Fenusinae + Blennocampinae) + (Belesinae + Caliroinae)) (ML = 59.6%), together forming a clade sister to Megabelesinae (ML = 99.7%). In the ML analyses of the 13 PCGs + rRNA dataset, it was recovered as sister to (((Fenusinae + Lycaotinae) + Blennocampinae) + (Belesinae + Caliroinae)) (ML = 76.5%), again forming a sister relationship with Megabelesinae (ML = 100%) (Figure S2).

BI analyses produced broadly similar but more strongly supported relationships. In the 13 PCGs dataset, the Tenthredininae + Allantinae lineage was supported as sister to ((Fenusinae + Blennocampinae) + ((Megabelesinae + Caliroinae) + Belesinae)) (PP = 1). In the 13 PCGs + rRNA dataset, it was supported as sister to (((Fenusinae + Blennocampinae) + Lycaotinae) + ((Megabelesinae + Caliroinae) + Belesinae)) (PP = 1).

Several subfamilial relationships were stable across all analyses. The Selandriinae group consistently formed the clade (((Selandriinae + Strongylogasterinae) + Rocaliinae) + Dolerinae) with strong support (PP = 1, ML = 99.8%). Similarly, the Nematinae group was consistently recovered as ((Hoplocampinae + Nematinae) + Cladiinae) (PP = 1, ML = 100%) (Figure S2).

In summary, two alternative basal placements for Tenthredinidae were recovered. Analyses based on the 13 PCGs dataset supported the Selandriinae group as the basal lineage, whereas analyses incorporating rRNA data supported the Nematinae group as basal, highlighting the sensitivity of deep-level relationships to dataset composition.

### 3.3. Divergence Time Estimations at Subfamily Level

**Figure 3:**
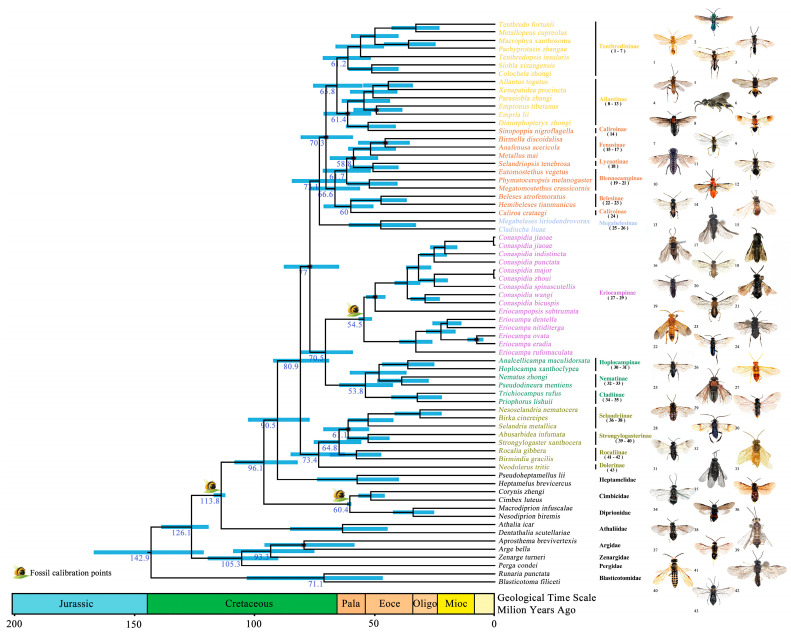
Dated phylogeny of Tenthredinidae estimated using the MCMCtree approach based on the maximum likelihood (ML) topology inferred from the 13 protein-coding genes (13PCGs). The geological time scale (in million years ago, Ma) is displayed at the bottom. Blue bars at the nodes represent the 95% highest posterior density (HPD) intervals for divergence times. Sawfly symbols mark three fossil calibration points. Black dots on the nodes indicate branches with bootstrap support values below 90. Representative images of adult specimens from 43 genera within the ingroup are shown on the right, with corresponding numbers in parentheses under each subfamily.

The divergence and diversification time estimates for Tenthredinidae were inferred from the ML analysis of a 13-PCG dataset using a secondary-calibrated relaxed molecular clock. According to the dated phylogeny, the stem-group age of Tenthredinidae was estimated at 90.51 Ma (77.01–102.51 Ma) in the Late Cretaceous, corresponding to the split between Heptamelidae and Tenthredinidae (Figure 3). The diversification of Tenthredinidae occurred during the Late Cretaceous. The earliest divergent lineage within the family was the fern sawfly clade, comprising ((Selandriinae + Strongylogasterinae) + Rocaliinae) + Dolerinae, with an estimated age of 73.37 Ma (62.01–84.99 Ma). The stem age of *Conaspidia*, *Eriocampopsis*, and *Eriocampa* was estimated at 70.47 Ma (58.77–81.09 Ma), corresponding to the boundary between the Late Cretaceous and the Paleocene. Among them, *Eriocampa* diverged first, with an estimated divergence time of 54.55 Ma (50.91–56.63 Ma) in the early Eocene, while *Eriocampopsis* and *Conaspidia* split during the early Eocene at 49.85 Ma (45.83–53.40 Ma). The diversification of *Conaspidia* occurred near the boundary of the Eocene and Oligocene, at approximately 36.54 Ma (31.03–41.85 Ma).

## 4. Discussion

### 4.1. Mitogenome Structure of Conaspidia, Eriocampopsis, and Eriocampa

The present study provides the first systematic analysis of mitochondrial genomes from 15 species representing the genera *Conaspidia*, *Eriocampopsis*, and *Eriocampa*, and summarizes their structural features at the subfamily level. The results revealed that these mitochondrial genomes are overall highly conserved in size, nucleotide composition, and codon usage (Figure S1, Table S5), with genome sizes falling within the ranges reported for other Tenthredinidae and ‘Symphyta’ species [39]. In addition, consistent with the known rearrangement hotspots in Hymenopteran mitochondrial genomes [40,41], frequent rearrangements were observed in the IQM, ARNS1EF, and WCY gene clusters (Figure 1), which may be associated with abnormal replication initiation or illegitimate recombination events [42,43]. Among these, tRNA rearrangements were particularly prominent, showing genus-specific characteristic patterns: for example, the *cox3*(+)–*trnG*(+)–*nad3*(+) block underwent remote inversion in *Conaspidia*; rearrangements within the *trnI*(+)–*trnQ*(-)–*trnM*(+) cluster occurred in *Eriocampa*; and complex rearrangements involving multiple tRNAs upstream of *nad2* were identified in *Eriocampopsis*. These features not only support the frequent tRNA gene rearrangements reported in ‘Symphyta’ [39,44,45,46,47,48], but may also provide potential molecular synapomorphies for the corresponding lineages.

### 4.2. Phylogenetic Placement of Conaspidia, Eriocampopsis, and Eriocampa and Recognition of Eriocampinae stat. nov.

In this study, 55 samples representing 43 genera across 15 subfamilies of Tenthredinidae were analyzed to explore the family’s internal phylogenetic relationships, integrating several major classification systems proposed since the establishment of the modern concept of Tenthredinidae. The results indicate that Athaliidae is positioned outside Tenthredinidae, Heptameliidae, Cimbicidae, and Diprionidae, consistent with the proposal by Niu et al. [12] (2022) to elevate Athaliini to the family level (Athaliidae). All analyses support the conclusion of Malm & Nyman (2015) [8] that *Heptamelus* and *Pseudoheptamelus* should be recognized as the family-level taxon Heptameliidae, a result also supported by Boevé (2013) [49]. 

Phylogenetic analyses further suggest that Tenthredinidae can be divided into five major lineages and one independent branch: the Selandriinae group (Selandriinae, Strongylogasterinae, Rocaliinae, and Dolerinae); the Nematinae group (Cladiinae, Nematinae, and Hoplocampinae); the *Eriocampa* group; Megabelesinae; the Blennocampinae group (Caliroinae, Belesinae, Blennocampinae, Lycaotinae, and Fenusinae); and the Tenthredininae group (Allantinae and Tenthredininae). Within Tenthredinidae, the Selandriinae group occupies the basal position, with the relationships resolved as (((Selandriinae + Strongylogasterinae) + Rocaliinae) + Dolerinae). This result is consistent with Benson (1938) [15], who first suggested a close relationship between Dolerinae and Selandriinae, and also supports Taeger’s (2010) [5] conclusion that Dolerinae is an internal lineage within a broadly defined Selandriinae. However, our study does not support Taeger’s (2010) [5] view that Selandriinae is not the most basal lineage of Tenthredinidae. 

The Nematinae group, which has often been considered the basal lineage of Tenthredinidae, was recovered in our analyses as the sister group of the *Eriocampa* group, together forming the second basal lineage of the Tenthredinidae. Their internal relationships were consistently resolved across all analyses as ((Hoplocampinae + Nematinae) + Cladiinae). By contrast, the relative positions of subfamilies within the Blennocampinae group (Fenusinae, Blennocampinae, Lycaotinae, Belesinae, and Caliroinae) remain unstable. Given the current taxon sampling, it is premature to propose systematic revisions of these subfamilies, and more comprehensive phylogenetic analyses incorporating broader generic and species representation will be required.

Notably, the *Eriocampa* group (*Conaspidia*, *Eriocampopsis*, and *Eriocampa*) was consistently recovered as a strongly supported monophyletic clade across all analyses, supporting its validity as an independent systematic unit. Accordingly, we recognize Eriocampinae Rohwer, 1911 stat. nov. to accommodate the *Eriocampa* group. The type genus of the subfamily is *Eriocampa* Hartig, 1837.

Recent phylogenomic studies based on nuclear genes and ultraconserved elements (UCEs) provide an important framework for evaluating the robustness of mitochondrial-based phylogenies. In particular, Wutke et al. (2024) [9] recovered *Conaspidia* and *Eriocampa* as sister lineages in their nuclear phylogeny, a result that is fully congruent with our mitochondrial analyses. However, this relationship was not discussed in detail nor translated into a formal taxonomic hypothesis in that study, as their primary focus was on higher-level relationships within ‘Symphyta’. In addition, nuclear-based phylogenies generally show limited resolution or reduced support for the placement of Eriocampini relative to other major tenthredinid lineages.

Although the external morphology of *Eriocampa* and *Conaspidia* differs markedly, particularly in head structure and male genitalia, the congruent recovery of these genera as sister lineages in both mitochondrial and nuclear datasets indicates that their close relationship is robust. The partial incongruence between mitochondrial and nuclear topologies at deeper nodes may reflect differences in evolutionary rates, lineage-specific rate heterogeneity, or biological processes such as incomplete lineage sorting or ancient introgression. Taken together, the agreement at this critical node, combined with mitogenomic rearrangements and morphological evidence, supports the recognition of Eriocampinae stat. nov. 

### 4.3. Morphology of Eriocampinae

Major morphological comparisons between Eriocampinae and other subfamilies of Tenthredinidae:

Phylogenetic trees reconstructed from both mitochondrial genomes and nuclear genes/UCEs strongly support *Eriocampa* and *Conaspidia* as forming a monophyletic group that is distinct from all other subfamilies of Tenthredinidae. Assigning Eriocampinae to any existing subfamily would violate the principle of monophyly and lacks any supporting morphological evidence. Therefore, the establishment of Eriocampinae as an independent subfamily is necessary.

In traditional classifications of Tenthredinidae, *Eriocampa* and *Conaspidia* have consistently been placed in different subfamilies, and no previous studies have reported shared morphological characters between these two taxa. The comparative morphological study of the present subfamily was conducted only after molecular phylogenetic analyses supported its monophyly, followed by a comprehensive comparative assessment. In the following morphological comparisons, all 19 subfamilies of Tenthredinidae recognized by Wei (2024) [13] were examined, encompassing more than 340 genera and species.

Higher-level systematics of Tenthredinidae is primarily based on morphological characters such as head configuration, the structure of the mandibles and antennae, the pronotum, mesopleuron, and metanotum, and venation, as well as the ovipositor and penis valve.

**Figure 4:**
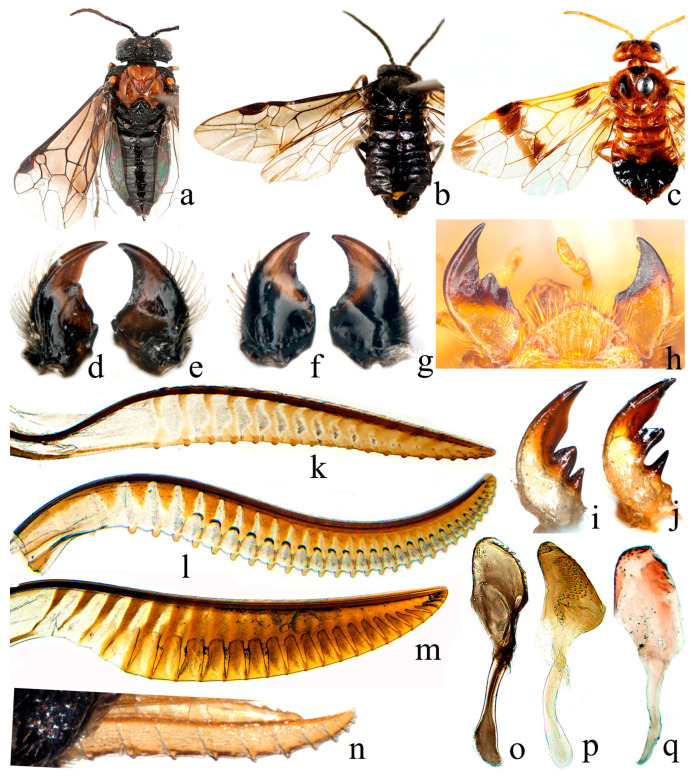
Eriocampinae. (**a**–**c**), adult females, dorsal view; (**d**,**f**,**i**,**j**), left mandible; (**e**,**g**), right mandible; (**h**). mandibles; (**k**–**n**), lancet; (**o**–**q**), penis valve; (**a**,**d**,**e**,**k**,**o**), *Eriocampa nitiditerga* Wei, 2025; (**b**,**f**,**g**,**n**), *Eriocampopsis acuata* Wei & Niu, 2010; (**c**,**h**,**q**), *Conopid guttata* (Matsumura, 1912); (**i**,**p**). *C. jiaoae* Wei, 2025; (**j**). *C. sikkimensis* Konow, 1898; (**l**). *C. bicuspis* Malaise, 1945; (**m**). *C. wangi* Wei, 2015.

Antennae: The antennae of all genera in Eriocampinae represent the basic antennal type of Tenthredinidae and lack distinctive diagnostic features (Figure 4a–c).

Head type: The head of *Eriocampa* differs from that of all other subfamilies of Tenthredinidae. Its compound eyes and clypeal configuration are similar to those of Allantinae, but the head behind the eyes is extremely narrow but distinctly convex, and the frontal carina is sharply developed, resembling certain taxa of Strongylogasterinae (Figure 4a). In contrast, the head of *Conaspidia* is very similar to that of well-developed members of Allantinae (Figure 4c).

Mandibles: The left and right mandibles of Eriocampinae are distinctly asymmetrical, with a multidentate left mandible and a simplified right mandible (Figure 4e,g), resembling the condition in well-developed Allantinae but clearly differing from other subfamilies. In *Eriocampa* and *Eriocampopsis*, the middle tooth of the left mandible is extremely broad and short, being almost indistinguishable, while the basal tooth is very small (Figure 4d,f). In contrast, *Conaspidia* exhibits well-developed and clearly differentiated middle and basal teeth on the left mandible (Figure 4h–j).

Thorax: In *Eriocampa*, the pronotum is extremely narrow and represents the primitive condition in Tenthredinidae. In *Conaspidia*, the anterior part of the pronotal groove is slightly expanded, resembling that of some primitive lineages of Allantinae. In all three genera of Eriocampinae, the ventral contact area of the propleura is relatively narrow, similar to that of some Allantinae taxa. The metascutellum of Eriocampinae is conspicuously transversely expanded, and the cenchri are small and widely separated. This condition is unique among Tenthredinidae and represents a highly specialized morphology. The mesepimeron in Eriocampinae is very broad, completely covering the metathoracic spiracle, with a strongly concave median portion. This morphology is similar to that observed in Dolerinae, Allantinae, and Tenthredininae, but differs from other subfamilies. However, the degree of expansion and posterior projection of the mesepimeron in Eriocampinae is more pronounced than in Dolerinae, Allantinae, and Tenthredininae. The metapostnotum of Eriocampinae is extremely narrow and strongly inclined ventrally, representing a relatively inconspicuous but diagnostic character of this subfamily.

Venation: The wing venation of Eriocampinae generally conforms to the basic pattern of Tenthredinidae but exhibits certain specializations. The anal cell of the forewing is complete and bears an oblique crossvein beyond the middle, a condition similar to that of Allantinae, Belesinae, Caliroinae, Dolerinae, and Hoplocampinae, but different from other subfamilies. Two types of venational configurations are observed around the 1M cell. In *Eriocampa*, the pattern is somewhat similar to that of Blennocampinae (Figure 4a,b), whereas in *Conaspidia,* it more closely resembles that of the Allantinae genera *Adamas* and *Dimorphopteryx* (Figure 4c). In the hind wing, both the Rs cell and the M cell are closed, representing the primitive condition in Tenthredinidae. This pattern is similar to that found in Nematinae, Cladiinae, Dolerinae, Selandriinae, Rocaliinae, Strongylogasterinae, and most genera of Tenthredininae.

Ovipositor: The lancet of Eriocampinae is narrow and elongated, with no significant sclerotization. The radix is very short, and the lamnium bears relatively narrow annuli with numerous annular sutures (Figure 4k–n). This condition is similar to that observed in Tenthredininae and Allantinae but differs from other subfamilies.

Penis valve: The penis valve of Eriocampinae lacks a lateral-apical hook, has no dorsal or ventral marginal teeth (*Eriocampa* has a row of large submarginal teeth), and is devoid of various spurs or lateral lobes (Figure 4o–q). Its configuration most resembles that of Tenthredininae and Megabelesinae but differs markedly from other subfamilies.

Based on a comprehensive comparison of morphological characters across all subfamilies of Tenthredinidae, the morphology of Eriocampinae is most similar to Allantinae and Tenthredininae. Specifically, the venation, mandibles, and head type are more similar to those of Allantinae, whereas the configuration of the penis valve is closer to that of Tenthredininae.

Diagnostic characters of Eriocampinae:

A. The mesepimeron is strongly and triangularly expanded, with a broad concave median portion. B. The metapostnotum is narrow and steep, almost perpendicular to the body axis. C. The metascutellum is transversely expanded, with small and widely separated cenchri. D. The mandibles are markedly asymmetrical, with the left mandible tridentate and the right mandible unidentate. E. The antennae are simple and filiform, with 9 segments; the second segment is longer than wide. F. The pronotum is narrow, and the ventral contact area of the propleura is narrow. G. In the forewing, vein 1M is longer than 1m-cu, and the two veins run approximately parallel; vein R+M is short; the anal cell is closed and bears an oblique crossvein beyond the middle. In the hind wing, both the Rs and M cells are closed, and the anal cell is petiolate. H. The ovipositor is long and weakly sclerotized; the radix is very short; the lamnium is long; and annular sutures are distinct and dense. I. The valviceps of the penis valve is simple, without any spur or lateral lobe, and lacks dorsal and ventral marginal teeth.

Among these characters, A–C may represent synapomorphies unique to this subfamily. Character D is generally similar to Allantinae, but with intergeneric differences. Character H is shared with Allantinae and Tenthredininae, while character I is shared with Tenthredininae.

### 4.4. Divergences of Eriocampinae

The dated phylogenetic tree obtained in this study further indicates that the divergence and diversification times of *Conaspidia*, *Eriocampopsis*, and *Eriocampa* broadly overlap with those of their known host plants, although considerable uncertainty exists around the precise timing. *Conaspidia* is endemic to East Asia, with 24 described species, and primarily feeds on *Kalopanax* and *Aralia* (Araliaceae) [19,50]. *Eriocampa* occurs in the Holarctic, with about 20 described species (more undescribed species are expected in East Asia), and utilizes *Alnus* and *Corylus* (Betulaceae) as well as *Juglans* (Juglandaceae) [51,52]. *Eriocampopsis* is a small genus endemic to East Asia with two described species, and the host plants remain uncertain [53].

From a phylogenetic perspective, Araliaceae belongs to Magnoliopsida (dicots)—Eudicots—Asterids—Apiales, occupying a relatively derived position within the asterids. This family represents a comparatively “young” and highly diversified lineage of eudicots [54], with crown ages estimated at approximately 49–73 Ma (Late Cretaceous–Eocene) [55]. Betulaceae likely originated in the Late Cretaceous (~70 Ma), with major subfamilies (Betuloideae and Coryloideae) diverging during the Paleocene [56]. Juglandaceae is estimated to have a stem-group age of ~78.7 Ma, with rapid diversification of its major tribes during the Paleocene–Eocene [57].

Overall, Araliaceae, Betulaceae, and Juglandaceae occupy relatively derived positions in the angiosperm phylogeny, representing highly diversified eudicot lineages rather than basal groups. While the divergence and diversification times of *Conaspidia* and *Eriocampa* broadly coincide with those of their host plants, these temporal overlaps should not be interpreted as direct evidence of strict coevolution. Other scenarios, such as host shifts onto already diversified plant lineages, cannot be excluded. We also note that the timing of diversification inferred from plant chronograms carries uncertainties arising from different calibration points, dating methods, and model assumptions, which may affect the apparent correspondence with insect divergence times.

## Data Availability

The data presented in this study are openly available in Science Data Bank at DOI: 10.57760/sciencedb.31736. The nomenclatural act for Eriocampinae has been registered in ZooBank (LSID: urn:lsid:zoobank.org:act:9A768C2E-2DE2-4981-8FD3-E37E1C806CB5).

## Supplementary Materials

The following supporting information can be downloaded at: https://www.mdpi.com/article/10.3390/biology15020202/s1, Figure S1: Relative Synonymous Codon Usage (RSCU) in Eriocampinae, Figure S2: Phylogenetic trees of Tenthredinidae reconstructed with 13PCGs using (a) ML, (b) BI; of 13PCGs + rRNA using (c) ML and (d) BI. For ML trees, the numbers at the nodes indicate bootstrap support and UF Boot support values, respectively (bootstrap support/UF Boot). For BI trees, the numbers at the nodes represent posterior probabilities. Table S1: Summary information of mitogenomes used in phylogenetic analyses, Table S2: Sample collection and accession information of the Eriocampinae, Table S3: Fossil calibration points used for divergence time estimation in MCMCtree analysis, Table S4: The mitochondrial genome base composition of the Eriocampinae, Table S5: Relative synonymous codon usage (RSCU) of the Eriocampinae mitogenomes. References [9,10,11,12,32,33,34,58,59,60,61,62,63,64,65,66,67,68,69,70,71] are cited in the supplementary materials.
