# Supplementary material for: Mitochondrial Phylogenomics of Tenthredinidae (Hymenoptera: Tenthredinoidea) Supports the Monophyly of Eriocampinae stat. nov
Source: Biology (Basel). 2026 Jan 22;15(2):202. doi: 10.3390/biology15020202 (PMC12837920; doi:10.3390/biology15020202)
Supplement: Supplementary file 1 [file biology-15-00202-s001.zip › biology-4086407 - Supplementary new.pdf]

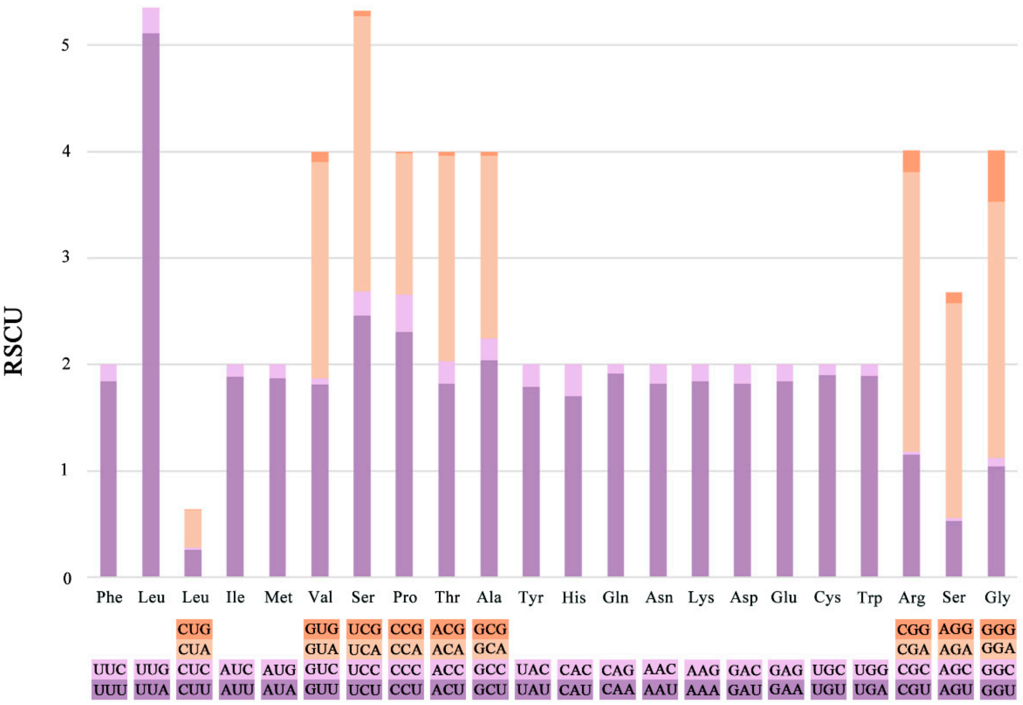

Figure S1 Relative Synonymous Codon Usage (RSCU) in Eriocampinae

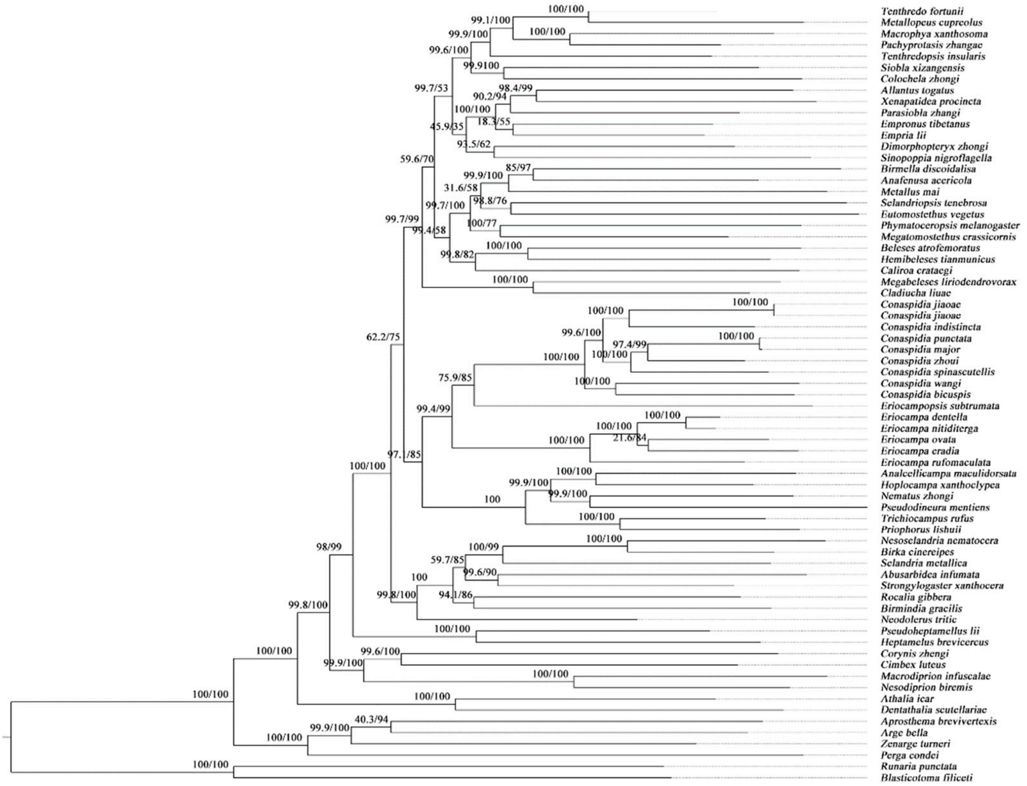

a.

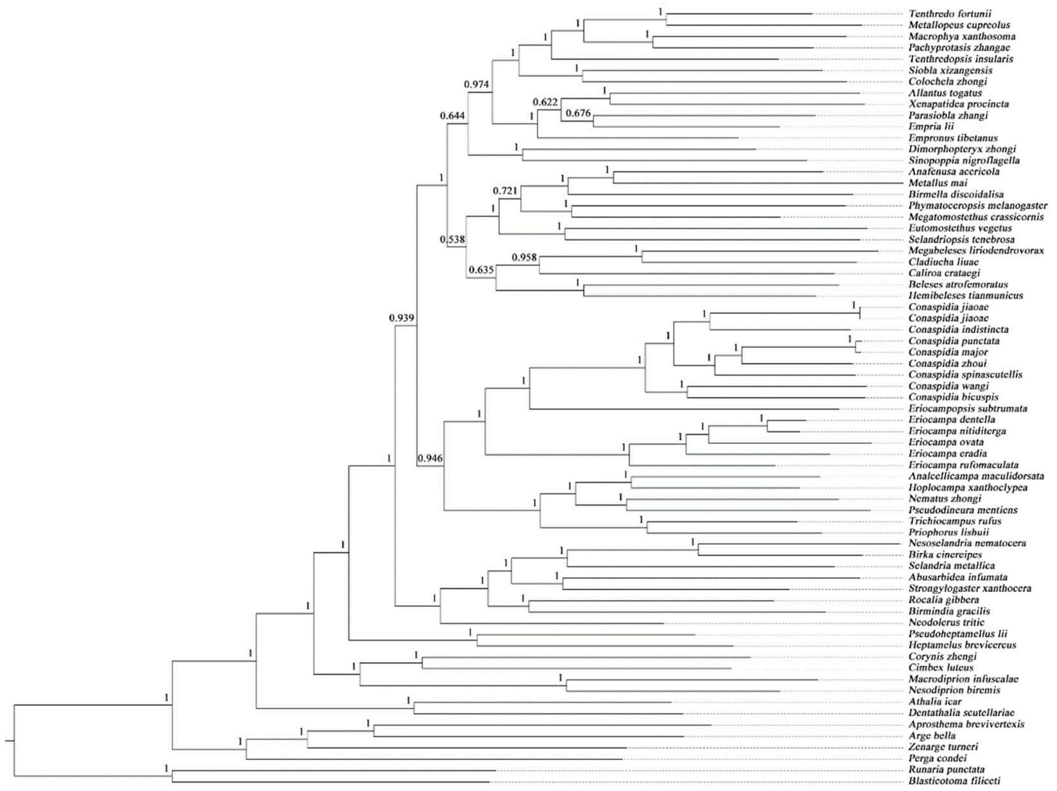
Tenthredo fortunii
Metallopeus cupreolus
Macrophya xanthosoma
Pachyprotasis zhangae
Tenthredopsis insularis
Siobla xizangensis
Colochela zhongi
Allantus togatus
Xenapatidea procincta
Parasiobla zhangi
Empria lii
Empronus tibetanus
Dimorphopteryx zhongi
Sinopoppia nigroflagella
Anafenusa acericola
Metallus mai
Birmella discoidalisa
Phymatoceropsis melanogaster
Megatomostethus crassicornis
Eutomostethus vegetus
Selandriopsis tenebrosa
Megabeleses liriodendrovorax
Cladiucha liuae
Caliroa crataegi
Beleses atrofemoratus
Hemibeleses tianmunicus
Conaspidia jiaoae
Conaspidia jiaoae
Conaspidia indistincta
Conaspidia punctata
Conaspidia major
Conaspidia zhoui
Conaspidia spinascutellis
Conaspidia wangi
Conaspidia bicuspis
Eriocampopsis subtrumata
Eriocampa dentella
Eriocampa nitiditerga
Eriocampa ovata
Eriocampa eradia
Eriocampa rufomaculata
Analcellicampa maculidorsata
Hoplocampa xanthoclypea
Nematus zhongi
Pseudodineura mentiens
Trichiocampus rufus
Priophorus lishuii
Nesoselandria nematocera
Birka cinereipes
Selandria metallica
Abusarbidea infumata
Strongylogaster xanthocera
Rocalia gibbera
Birmindia gracilis
Neodolerus tritic
Pseudoheptamellus lii
Heptamelus brevicercus
Corynis zhengi
Cimbex luteus
Macrodiprion infuscalae
Nesodiprion biremis
Athalia icar
Dentathalia scutellariae
Aprosthema brevivertexis
Arge bella
Zenarge turneri
Perga condei
Runaria punctata
Blasticotoma filiceti

b.

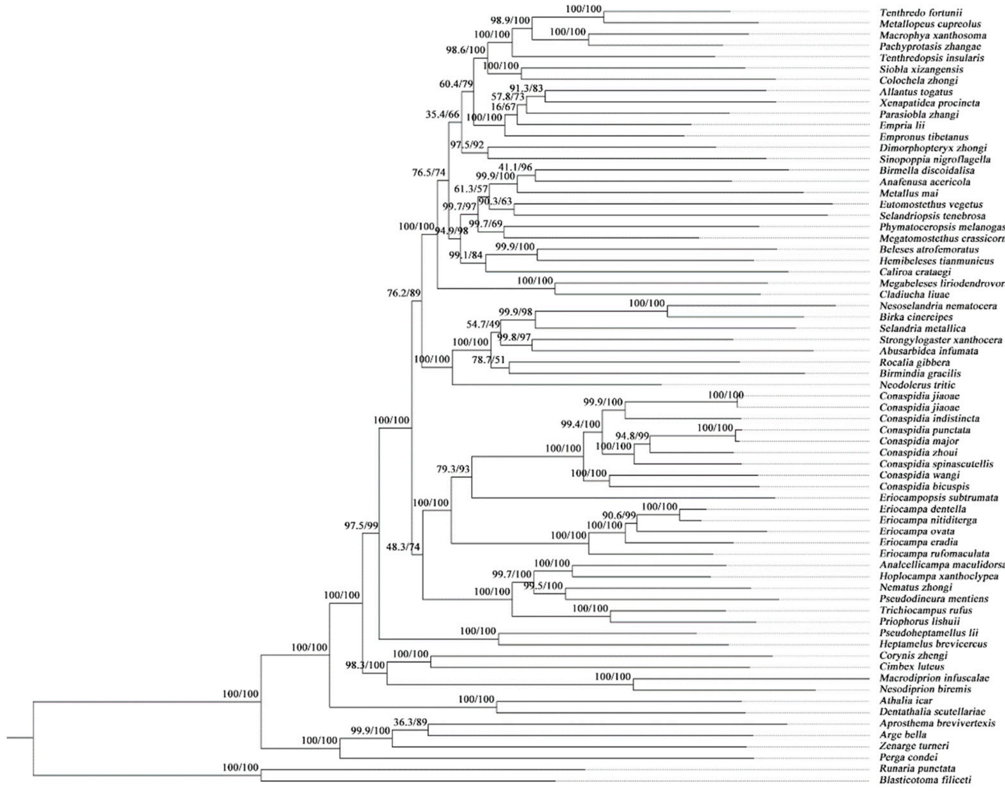
Tenthredo fortunii
Metallopeus cupreolus
Macrophya xanthosoma
Pachyprotasis zhangae
Tenthredopsis insularis
Siobla xizangensis
Colochela zhongi
Allantus togatus
Xenapatidea procincta
Parasiobla zhangi
Empria lii
Empronus tibetanus
Dimorphopteryx zhongi
Sinopoppia nigroflagella
Birmella discoidalisa
Anafenusa acericola
Metallus mai
Eutomostethus vegetus
Selandriopsis tenebrosa
Phymatoceropsis melanogaster
Megatomostethus crassicornis
Beleses atrofemoratus
Hemibeleses tianmunicus
Caliroa crataegi
Megabeleses liriodendrovorax
Cladiucha liuae
Nesoselandria nematocera
Birka cinereipes
Selandria metallica
Strongylogaster xanthocera
Abusarbidea infumata
Rocalia gibbera
Birmindia gracilis
Neodolerus tritic
Conaspidia jiaoae
Conaspidia jiaoae
Conaspidia indistincta
Conaspidia punctata
Conaspidia major
Conaspidia zhoui
Conaspidia spinascutellis
Conaspidia wangi
Conaspidia bicuspis
Eriocampopsis subtrumata
Eriocampa dentella
Eriocampa nitiditerga
Eriocampa ovata
Eriocampa eradia
Eriocampa rufomaculata
Analcellicampa maculidorsata
Hoplocampa xanthoclypea
Nematus zhongi
Pseudodineura mentiens
Trichiocampus rufus
Priophorus lishuii
Pseudoheptamellus lii
Heptamelus brevicercus
Corynis zhengi
Cimbex luteus
Macrodiprion infuscalae
Nesodiprion biremis
Athalia icar
Dentathalia scutellariae
Aprosthema brevivertexis
Arge bella
Zenarge turneri
Perga condei
Runaria punctata
Blasticotoma filiceti

c.

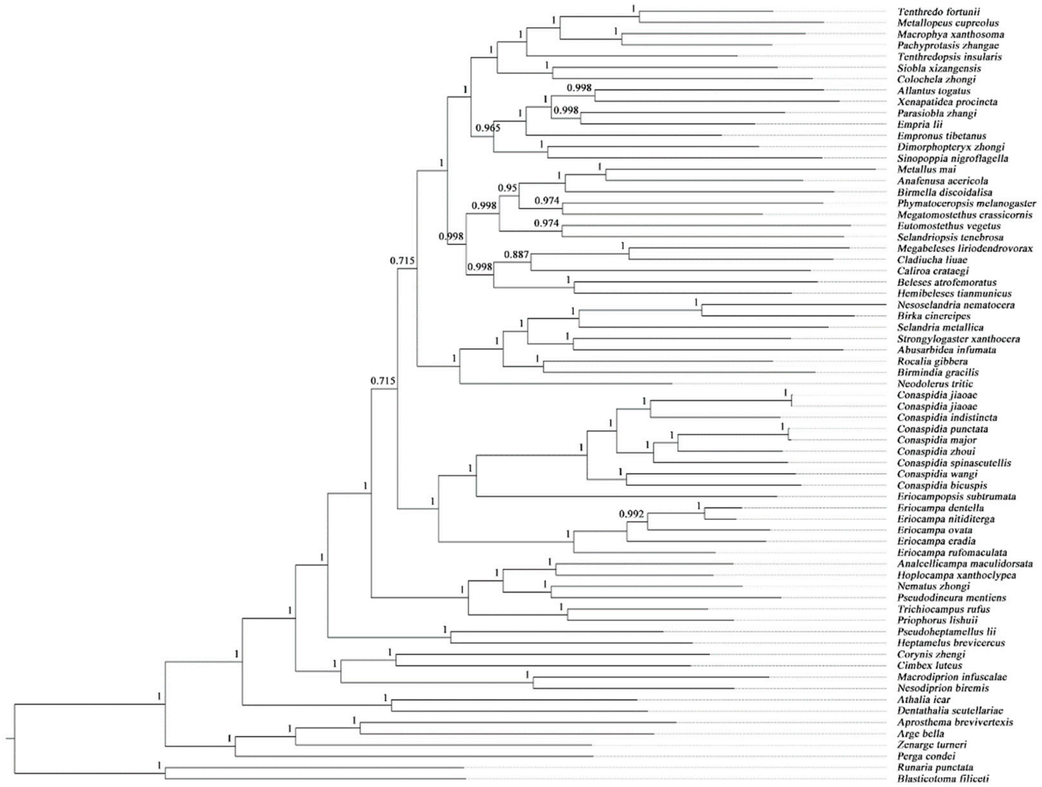

d.

Figure S2 Phylogenetic trees of Tenthredinidae reconstructed with 13PCGs using (a) ML, (b) BI; of 13PCGs + rRNA using (c) ML and (d) BI. For ML trees, the numbers at the nodes indicate bootstrap support and UF Boot support values, respectively (bootstrap support/UF Boot). For BI trees, the numbers at the nodes represent posterior probabilities.

Table S1 Summary information of mitogenomes used in phylogenetic analyses

	Family	Subfamily	Species	Accession Number	References	Location
Ingroup	Tenthredinidae	Eriocampinae	*Eriocampa dentella*	PX859821	This study	China: Hulunbuir, Inner Mongolia
			Eriocampa nitiditerga	PX859822	This study	China: Mt. Emei, Sichuan
			Eriocampa ovata	OK287350	[10]	Switzerland: Villa Luganese, Ticino
			Eriocampa eradia	PX859823	This study	China: Songpan, Sichuan
			Eriocampa rufomaculata	PX859824	This study	China: Shennongjia, Hubei
			Eriocampopsis subtrumata	PX859825	This study	China: Fusong, Jilin
			Conaspidia jiaoae	PX859826	This study	China: Tianshui, Gansu
			Conaspidia jiaoae	PX859827	This study	China: Qingshui, Gansu
			Conaspidia indistincta	PX859828	This study	China: Shennongjia, Hubei
			Conaspidia punctata	PX741068	This study	China: Mt. Taibai, Shaanxi
			Conaspidia major	PX859829	This study	China: Foping, Shaanxi
			Conaspidia spinascutellis	PX859830	This study	China: Shennongjia, Hubei
			Conaspidia wangi	MW415019	[58]	China: Jiangkou, Guizhou
			Conaspidia bicuspis	PX859831	This study	China: Shimen, Hunan
			Conaspidia zhoui	PX859832	This study	China: Mt. Gongga, Sichuan
		Cladiinae	*Trichiocampus rufus*	PX859833	This study	China: Chengde, Hebei
			Priophorus lishuii	PX859834	This study	China: Lishui, Zhejiang
		Nematinae	*Nematus zhongi*	PX859835	This study	China: Yizhang, Hunan
			Pseudodineura mentiens	SRR20665993	[9]	\
		Hoplocampinae	*Hoplocampa xanthoclypea*	PX859836	This study	China: Shanghe, Shandong
			Analcellicampa maculidorsata	PX859837	This study	China: Xiushui, Jiangxi
		Dolerinae	*Neodolerus tritici*	PX859838	This study	China: Xinan, Henan
		Rocaliinae	*Rocalia gibbera*	PX859839	This study	China: Mt. Emei, Sichuan

	Family	Subfamily	Species	Accession Number	References	Location
Ingroup	Tenthredinidae	Strongylogasterinae	*Birmindia gracilis*	PX859840	This study	China: Mt. Emei, Sichuan
			Strongylogaster xanthocera	MW324676	[59]	China: Neixiang, Henan
			Abusarbidea infumata	PX859841	This study	China: Sangzhi, Hunan
		Selandriinae	*Selandria metallica*	PX859842	This study	China: Shennongjia, Hubei
			Nesoselandria nematocera	PX859843	This study	China: Mt. Emei, Sichuan
			Birka cinereipes	PX859844	This study	Greece: Konitsa, Epirus
		Megabelesinae	*Megabeleses liriodendrovorax*	MW255939	[11]	China: Mt. Dawei, Henan
			Cladiucha liuae	PX859845	This study	China: Shaoguan, Guangdong
		Lycaotinae	*Selandriopsis tenebrosa*	PX859846	This study	Turkey: Sivas Cumhuriyet University Campus, Sivas
		Blennocampinae	*Phymatoceropsis melanogaster*	MZ265346	[11]	China: Chanyuan Temple, Zhejiang
			Megatomostethus crassicornis	MZ265345	[11]	China: Mt. Tianmu, Zhejiang
			Eutomostethus vegetus	MT663219	[60]	China: Mt. Lu, Jiangxi
		Fenusinae	*Birmella discoidalisa*	MF197548	[61]	China: Mt.Emei, Sichuan
			Metallus mai	MW255941	[11]	China: Mt. Lu, Jiangxi
			Anafenusa acericola	OR730576	[62]	China: Tai'an, Shandong
		Caliroinae	*Caliroa crataegi*	PX859847	This study	China: Longnan, Gansu
			Sinopoppia nigroflagella	MW487927	[63]	China: Lanxi, Zhejiang
		Belesinae	*Beleses atrofemoratus*	MZ265347	[11]	China: Mt. Qiyun, Hunan
			Hemibeleses tianmunicus	MZ265344	[11]	China: Chanyuan Temple, Zhejiang
		Allantinae	*Xenapatidea procincta*	MW487928	[11]	China: Kaishan Old Temple, Zhejiang
			Dimorphopteryx zhongi	OK329944	[10]	China: Foping, Shaanxi
			Empria lii	MW632124	[11]	China: Dalongtan, Hubei
			Allantus togatus	MW464859	[64]	SPAIN: Serra, Valencia

	Family	Subfamily	Species	Accession Number	References	Location
Ingroup	Tenthredinidae	Allantinae	*Empronus tibetanus*	MZ265343	[11]	China: Songlinkou, Tibet Autonomous Region
			Parasiobla zhangi	MZ461490	[65]	China: Jiuzhaigou, Sichuan
		Tenthredininae	*Tenthredo fortunii*	PX859848	This study	China: Guidong, Hunan
			Colochela zhongi	MT702984	[66]	China: Mt. Taibai, Shaanxi
			Siobla xizangensis	MN562486	[67]	China: Motuo, Tibet Autonomous Region
			Metallopeus cupreolus	PX859849	This study	China: Mt. Emei, Sichuan
			Macrophya xanthosoma	PX859850	This study	China: Mt. Huping, Hunan
			Pachyprotasis zhangae	PX859851	This study	China: Lushi, Henan
			Tenthredopsis insularis	PX859852	This study	China: Mt. Tianmu, Zhejiang
Outgroup	Blasticotomidae	\	*Runaria punctata*	ON808427	[12]	China: Foping, Shaanxi
		\	*Blasticotoma filiceti*	ON840091	[12]	RU: Vrangel, Nakhodka
	Pergidae	\	*Perga condei*	AY787816	[68]	\
	Zenargidae	\	*Zenarge turneri*	SRR20666055	[9]	\
	Argidae	Arginae	*Arge bella*	MF287761	[69]	China: Mt. Qiyun, Hunan
		Sterictiphorinae	*Aprosthema brevivertexis*	PX859853	This study	China: Mt. Lu, Jiangxi
	Athaliidae	Athaliinae	*Athalia icar*	MN527306	[70]	China: Panan, Zhejiang
			Dentathalia scutellariae	ON808426	[12]	China: Qingshui, Gansu
	Diprionidae	Diprioninae	*Nesodiprion biremis*	ON964465	[12]	China: Mt. Mufu, Hunan
			Macrodiprion infuscalae	PX859854	This study	China: Youxi, Fujian
	Cimbicidae	Cimbicinae	*Cimbex luteus*	MW136447	[71]	China: Nanguan, Jilin
		Corynidinae	*Corynis zhengi*	OL549451	Unpublished	China: Menyuan, Qinghai
	Heptamelidae	Heptameliinae	*Heptamelus brevithecus*	MW632128	[11]	China: Mt. Yun, Hunan
			Psedoheptamellus lii	MW632128	[12]	China: Mt. Yun, Hunan

Table S2 Sample collection and accession information of the Eriocampinae

Species	Location	Longitude	Latitude	Altitude	Collection Date
Eriocampa eradia	China: Songpan, Sichuan	E.103°55'48"	N.32°46'56"	2703 m	2024-07-18
Conaspidia bicuspis	China: Shimen, Hunan	E.110°33′14″	N.30°0′50″	569 m	2021-04-27
Conaspidia wangi	China: Jiangkou, Guizhou	E.108°49′12″	N.27°40′48″	378 m	2018-09-08
Conaspidia major	China: Foping, Shaanxi	E.107°51′14″	N.33°41′5″	2115 m	2017-06-17
Conaspidia punctata	China: Mt. Taibai, Shaanxi	E.107°51′34″	N.34°0′34″	1815 m	2017-06-19
Eriocampopsis subtrumata	China: Fusong, Jilin	E.127°47′13″	N.42°30′40″	758 m	2015-05-24
Conaspidia jiaoae	China: Qingshui, Gansu	E.106°19′42″	N.34°49′33″	1595 m	2020-07-19
Conaspidia spinascutellis	China: Shennongjia, Hubei	E.110°27′29″	N.31°41′57″	2092 m	2022-07-03
Conaspidia jiaoae	China: Tianshui, Gansu	E.105°43'04"	N.34°28'12"	1510 m	2024-08-27
Eriocampa ovata	Switzerland: Villa Luganese, Ticino	E.9°01′29″	N.46°03′40″	2000 m	2009-05-24
Eriocampa rufomaculata	China: Shennongjia, Hubei	E.110°21′36″	N.31°25′12″	1516 m	2022-07-08
Conaspidia indistincta	China: Shennongjia, Hubei	E.110°40′52″	N.31°45′16″	1081 m	2022-07-01
Eriocampa dentella	China: Hulunbuir, Inner Mongolia	E.122°44'48"	N.48°00'36"	315 m	2021-04-01
Eriocampa nitiditerga	China: Mt. Emei, Sichuan	E.103°20′14″	N.29°31′22″	3071 m	2011-06-26
Conaspidia zhoui	China: Mt. Gongga, Sichuan	E.102°03′42″	N.29°48′19″	2754 m	2013-07-02

Table S3 Fossil calibration points used for divergence time estimation in MCMCtree analysis

Fossil taxon	Maximum age (Ma)	Minimum age (Ma)	Reference
Palaeathalia laiyangensis	125	113	[32]
Cenocimbex menatensis	61.66	59.24	[33]
Eriocampa tulameenensis	56	47.8	[34]

Table S4 The mitochondrial genome base composition of the Eriocampinae

Species	Length(bp)	A%	C%	G%	T%	A+T%	AT-Skew	GC-Skew
Eriocampa eradia	15105	42.6	12.9	7.8	36.7	79.3	0.0744	-0.2464
Conaspidia bicuspis	15730	42.1	11	7	39.9	82	0.0268	-0.2222
Conaspidia wangi	15924	41.2	11.9	7.7	39.2	80.4	0.0249	-0.2143
Conaspidia major	15327	41.7	11.5	7.5	39.3	81	0.0296	-0.2105
Conaspidia punctata	15834	41.7	11.3	7.4	39.6	81.3	0.0258	-0.2086
Eriocampopsis subtrumata	15182	43.2	11.3	7.4	38.1	81.3	0.0627	-0.2086
Conaspidia jiaoae	15221	41.9	11.7	7.7	38.8	80.7	0.0384	-0.2062
Conaspidia spinascutellis	16163	42	11.3	7.5	39.2	81.2	0.0345	-0.2021
Conaspidia jiaoae	15250	41.9	11.6	7.7	38.8	80.7	0.0384	-0.2021
Eriocampa ovata	15083	42.9	11.9	7.9	37.3	80.2	0.0698	-0.2020
Eriocampa rufomaculata	15208	43	11.5	7.8	37.7	80.7	0.0657	-0.1917
Conaspidia indistincta	15208	41.4	10.5	7.4	40.7	82.1	0.0085	-0.1732
Eriocampa dentella	15095	42.9	11	7.8	38.3	81.2	0.0567	-0.1702
Eriocampa nitiditerga	15104	43	10.9	7.8	38.3	81.3	0.0578	-0.1658
Conaspidia zhoui	15317	41.9	10.3	7.4	40.4	82.3	0.0182	-0.1638

Table S5 Relative synonymous codon usage (RSCU) of the Eriocampinae mitogenomes

**Amino**	**Codon**	**NO.**	**RSCU**	**Amino**	**Codon**	**NO.**	**RSCU**
Phe	UUU	351.3	1.84	Tyr	UAU	148.8	1.79
	UUC	30.6	0.16		UAC	17.5	0.21
Leu	UUA	504.6	5.11	End	UAA	0	0
	UUG	23.3	0.24		UAG	0	0
Leu	CUU	26.1	0.26	His	CAU	56.7	1.7
	CUC	1.8	0.02		CAC	10.2	0.3
	CUA	34.9	0.35	Gln	CAA	61.5	1.91
	CUG	1.2	0.01		CAG	3	0.09
Ile	AUU	393.3	1.88	Asn	AAU	224.3	1.82
	AUC	24.6	0.12		AAC	22.1	0.18
Met	AUA	289.7	1.87	Lys	AAA	112.6	1.84
	AUG	19.8	0.13		AAG	9.9	0.16
Val	GUU	60.5	1.81	Asp	GAU	55.6	1.82
	GUC	2	0.06		GAC	5.4	0.18
	GUA	68.1	2.03	Glu	GAA	67	1.84
	GUG	3.3	0.1		GAG	5.7	0.16
Ser	UCU	107.6	2.46	Cys	UGU	28.9	1.9
	UCC	10	0.23		UGC	1.5	0.1
	UCA	113	2.58	Trp	UGA	88.1	1.89
	UCG	2.1	0.05		UGG	5.1	0.11
Pro	CCU	71.3	2.31	Arg	CGU	13.7	1.15
	CCC	10.7	0.35		CGC	0.3	0.03
	CCA	40.9	1.32		CGA	31.3	2.63
	CCG	0.7	0.02		CGG	2.3	0.2
Thr	ACU	71.9	1.82	Ser	AGU	23.3	0.53
	ACC	8.4	0.21		AGC	1.1	0.03
	ACA	76.3	1.93		AGA	88.4	2.02
	ACG	1.4	0.04		AGG	4.4	0.1
Ala	GCU	54.5	2.04	Gly	GGU	47.1	1.04
	GCC	5.7	0.21		GGC	3.4	0.08
	GCA	45.7	1.71		GGA	108.8	2.41
	GCG	1	0.04		GGG	21.5	0.48

References

9. Wutke, S.; Blank, S.M.; Boevé, J.L.; Faircloth, B.C.; Koch, F.; Linnen, C.R.; Malm, T.; Niu, G.; Prous, M.; Schiff, N.M.; et al. Phylogenomics and biogeography of sawflies and woodwasps (Hymenoptera, Symphyta). *Mol. Phylogenet. Evol.* 2024, *199*, Article 108144.
10. Liu, M.; Li, M.; Wei, M.; Li, Z. The complete mitochondrial genome of *Eriocampa ovata* Linne, 1760 (Hymenoptera: Tenthredinidae) and phylogenetic analysis. *Mitochondrial DNA Part B* **2022**, *7*, 1789–1791.
11. Niu, G.; Jiang, S.; Doğan, Ö.; Korkmaz, E.M.; Budak, M.; Wu, D.; Wei, M. Mitochondrial phylogenomics of Tenthredinidae (Hymenoptera: Tenthredinoidea) supports the monophyly of Megabelesesinae as a subfamily. *Insects.* **2021**, *12(6)*, 495.
12. Niu, G.; Budak, M.; Korkmaz, E.M.; Doğan, Ö.; Nel, A.; Wan, S.; Cai, C.; Jouault, C.; Li, M.; Wei, M. Phylogenomic analyses of the Tenthredinoidea support the familial rank of Athaliidae (Insecta, Tenthredinoidea). *Insects*. 2022, *13(10)*, 858.
32. Zhang J. New data of the Mesozoic fossil insects from Laiyang in Shandong. Geol. Shandong 1985, 1, 23–39.
33. Nel A. New and poorly known Cenozoic sawflies of France (Hymenoptera: Tenthredinoidea, Pamphilioidea). Dtsch. Entomol. Z. 2004, 51(2), 253–269.
34. Rice, H. Two Tertiary sawflies (Hymenoptera-Tenthredinidae) from British Columbia. Geol. Surv. Can. Pap. 1968, 67-59, 1–21.
58. Yang, H.; Lu, Z.; Wei, M.; Niu, G. The complete mitochondrial genome of *Conaspidia wangi* Wei, 2015 (Hymenoptera: Tenthredinidae) and its phylogenetic analysis. *Mitochondrial DNA Part B* 2021, *6*, 2188–2190.
59. Liu, Y.; Wei, M.; Niu, G. The First Mitochondrial Genome of a Fern Sawfly, *Strongylogaster xanthocera* Stephens, 1835 (Hymenoptera: Tenthredinidae). *Mitochondrial DNA Part B* 2021, *6*, 902–904.
60. Li, Y.; Wei, M.; Liu, J.; Niu, G. Characterization of the Mitochondrial Genome of *Eutomostethus vegetus* Konow, 1898 (Hymenoptera: Tenthredinidae) and Phylogenetic Analysis. *Mitochondrial DNA Part B* 2020, *5*, 3033–3034.
61. Wu, R.; Wei, M.; Liu, M.; Niu, G. Advancement in Sequencing the Mitochondrial Genome of *Birmella discoidalisa* Wei, 1994 (Hymenoptera: Tenthredinidae) and the Phylogenetic Classification of Fenusini. *Mitochondrial DNA Part B* 2019, *4*, 4100–4101.
62. Tan, B.; Fu, H.; Niu, G.; Wei, M. A New Species of Sinoscolia Wei & Nie (Hymenoptera: Tenthredinidae) Mining Leaves of *Pterocarya stenoptera*. *Sci. Silvae Sin.* 2025, *61(1)*, 159–165.
63. Wan, S.; Wei, M.; Niu, G. The Complete Mitochondrial Genome Sequence of *Sinopoppia nigroflagella* Wei, 1997 (Hymenoptera: Tenthredinidae) Reveals a New Gene Order. *Mitochondrial DNA Part B* 2021, *6*, 999–1000.
64. Yang, J.; Sun, Z.; Wei, M.; Niu, G. The Complete Mitochondrial Genome of *Allantus togatus* (Panzer, 1801), in View of Possible Cryptic Species. *Mitochondrial DNA Part B* 2021, *6*, 1114–1115.
65. Xu, M.; Tan, B.; Wei, M.; Niu, G. The Complete Mitochondrial Genome Sequence of *Taxonus zhangi* Wei, 1997 (Hymenoptera: Tenthredinidae) with Phylogenetic Analysis. *Mitochondrial DNA Part B* 2021, *6*, 3188–3189.
66. Wu, D.; Wang, H.; Wei, M.; Niu, G. The Nearly Complete Mitochondrial Genome of *Colochela zhongi* Wei, 2016 (Hymenoptera: Tenthredinidae) and Phylogenetic Analysis. *Mitochondrial DNA Part B* 2020, *5*, 3341–3342.

67. Luo, X.; Wei, M.; Niu, G. Nearly Complete Mitochondrial Genome of *Siobla xizangensis* Xiao, Huang & Zhou, 1988 (Hymenoptera: Tenthredinidae) and Phylogenetic Analysis. *Mitochondrial DNA Part B* 2019, *4*, 4102–4103.
68. Castro, L.R.; Dowton, M. The Position of the Hymenoptera within the Holometabola as Inferred from the Mitochondrial Genome of *Perga condei* (Hymenoptera: Symphyta: Pergidae). *Mol. Phylogenetics Evol.* 2005, *34*, 469–479.
69. Du, S.; Niu, G.; Nyman, T.; Wei, M. Characterization of the Mitochondrial Genome of *Arge bella* Wei & Du Sp. Nov. (Hymenoptera: Argidae). *PeerJ* 2018, *6*, e6131.
70. He, H.; Niu, G.; Zhang, B.; Wei, M. The Complete Mitochondrial Genome of *Athalia proxima* (Hymenoptera: Tenthredinidae) and Phylogenetic Analysis. *Mitochondrial DNA Part B* 2019, *4*, 3868–3869.
71. Yan, Y.; Li, K.; Liu, S.; Niu, G.; Wei, M. The Complete Mitochondrial Genome of *Cimbex luteus* (Hymenoptera: Cimbicidae) and Phylogenetic Analysis. *Mitochondrial DNA Part B* 2021, *6*, 2031–2032.
